# Effects of blood glucose level on 18F-FDG uptake for PET/CT in normal organs: A systematic review

**DOI:** 10.1371/journal.pone.0193140

**Published:** 2018-02-27

**Authors:** Clarice Sprinz, Stephan Altmayer, Matheus Zanon, Guilherme Watte, Klaus Irion, Edson Marchiori, Bruno Hochhegger

**Affiliations:** 1 Department of Nuclear Medicine, Hospital Mãe de Deus, Porto Alegre, Rio Grande do Sul, Brazil; 2 Department of Radiology, Irmandade Santa Casa de Misericórdia de Porto Alegre, Porto Alegre, Rio Grande do Sul, Brazil; 3 Department of Diagnostic Methods, Federal University of Health Sciences of Porto Alegre, Rio Grande do Sul, Brazil; 4 Department of Radiology, Manchester Royal Infirmary, Central Manchester University Hospitals, Manchester, United Kingdom; 5 Department of Radiology, Federal University of Rio de Janeiro, Rio de Janeiro, Rio de Janeiro, Brazil; Banner Alzheimer’s Institute, UNITED STATES

## Abstract

**Purpose:**

To perform a systematic review of the effect of blood glucose levels on 2-Deoxy-2-[18F]fluoro-D-glucose (18F-FDG) uptake in normal organs.

**Methods:**

We searched the MEDLINE, EMBASE and Cochrane databases through 22 April 2017 to identify all relevant studies using the keywords “PET/CT” (positron emission tomography/computed tomography), “standardized uptake value” (SUV), “glycemia,” and “normal.” Analysis followed the Preferred Reporting Items for Systematic Reviews and Meta-Analyses recommendations. Maximum and mean SUVs and glycemia were the main parameters analyzed. To objectively measure the magnitude of the association between glycemia and 18F-FDG uptake in different organs, we calculated the effect size (ES) and the coefficient of determination (*R*^2^) whenever possible.

**Results:**

The literature search yielded 225 results, and 14 articles met the inclusion criteria; studies included a total of 2714 (range, 51–557) participants. The brain SUV was related significantly and inversely to glycemia (ES = 1.26; *R*^2^ 0.16–0.58). Although the liver and mediastinal blood pool were significantly affected by glycemia, the magnitudes of these associations were small (ES = 0.24–0.59, *R*^2^ = 0.01–0.08) and negligible (*R*^2^ = 0.02), respectively. Lung, bone marrow, tumor, spleen, fat, bowel, and stomach 18F-FDG uptakes were not influenced by glycemia. Individual factors other than glycemia can also affect 18F-FDG uptake in different organs, and body mass index appears to be the most important of these factors.

**Conclusion:**

The impact of glycemia on SUVs in most organs is either negligible or too small to be clinically significant. The brain SUV was the only value largely affected by glycemia.

## Introduction

Radiolabelled 2-deoxy-2-[18F]fluoro-D-glucose (18F-FDG) is a widely used radiopharmaceutical for the evaluation of tumor metabolism by positron emission tomography/computed tomography (PET/CT). In clinical practice, images are analyzed qualitatively by visual comparison of the metabolism in lesions and in normal tissues, or semiquantitatively using standardized uptake values (SUVs) [1]. 18F-FDG uptake in normal tissues is frequently adopted as an internal standard for tracer uptake, used as a reference when assessing tumor treatment response with PET [2].

Many studies have suggested that the plasma glucose level has a major influence on SUVs, but no clear consensus on the real impact of glycemia on 18F-FDG uptake has been reached. According to the European [3] and the American [2] guidelines, blood glucose should be measured prior to PET examinations, and tumor and brain imaging should be rescheduled when values exceed 200 mg/dl and 160 mg/dl, respectively [4]. Ideally, the 18F-FDG uptake of commonly used background tissues, such as the liver and mediastinal blood pool, should show no variation due to glycemic fluctuations to minimize variability in the assessment of treatment response [5]. However, the rescheduling of examinations can be inconvenient both for patients and for nuclear medicine practices. Moreover, recent studies have demonstrated that even mild hyperglycemia (<160 mg/dl) may decrease cortical 18F-FDG uptake, simulating the pattern seen in neurodegenerative diseases, such as Alzheimer’s, in healthy subjects [6].

The aim of this study was to systematically review the effect of blood glucose level on 18F-FDG uptake in normal organs, especially the liver, mediastinal blood pool, and brain. The impacts of factors other than glycemia were also analyzed.

## Materials and methods

### Study design

This study was conducted following the Preferred Reporting Items for Systematic Reviews (PRISMA) guidelines as shown in S1 File [7]. Diagnostic tests, cohort, and cross-sectional studies were included and no language or age of study restrictions were set.

The Cochrane Central Register of Controlled Trials (CENTRAL), Science Direct, Web of Science, and PubMed/MEDLINE electronic databases were comprehensively searched with the following keywords: (1) “PET-CT” OR “PET/CT” AND (2) “standardized uptake value” AND (3) “glycemia” OR “blood glucose” AND (4) “normal” OR “health”. The search criteria are represented in the S2 File. All databases were searched through 22 April, 2017. The search strategy developed for MEDLINE was adapted for other databases. In addition, reference lists of selected articles were hand screened for potential relevant studies that could have been missed during the electronic database search. Field experts were also consulted during the research process. Duplicate references were removed.

Other exclusion criteria were: studies that used 18F-FDG PET/CT to evaluate exclusively pathological conditions; studies in which 18F-FDG-PET was performed without integrated CT (not PET/CT) and studies with others radiopharmaceuticals (not 18F-FDG).

### Study selection

Eligibility of the selected articles was determined in two phases. In phase 1, three authors (MZ, CP and SA) independently screened titles and abstracts identified in all electronic databases. The authors selected articles that appeared to meet the inclusion criteria based on their title and abstracts. In phase 2, the same authors (MZ, CP and SA) read the full text of all selected articles, filtering them according to the inclusion and exclusion criteria. Disagreements between authors were solved by consensus and, when a consensus was not reached, a fourth author (BH) made a final decision.

### Data extraction

Two of the authors (MZ and SA) collected all key information in each article such as authors, year of publication, country, samples, median ages, study design, SUV, glycemia, time between 18F-FDG administration and scanning, reference standard, methods, results, and main conclusions. A third author (CP) crosschecked all the collected data. If required, disagreements were solved by consensus and a fourth author (BH) made a final decision.

### Study quality assessment

Three reviewers (MZ, CP and SA) evaluated the study quality of all selected articles using a checklist for general observational studies adapted and used by the Agency for Healthcare Research and Quality (AHRQ)[8,9]. This assessment tool include a 11-item questionnaire to explore the quality of patient recruitment, outcome measurement, blinding of the observers and follow-up of patients. Due to the cross-sectional nature of the studies herein included, the last question on patient follow-up was not applicable. Each item of the checklist was scored with “yes,” “no,” “unclear,” or “not applicable”. Disagreements between reviewers were solved by consensus and the opinion of a fourth reviewer (BH) if necessary. Finally, study quality was not an exclusion criterion.

### Summary measure

Maximum and mean standard uptake value (SUV_max_/SUV_mean_), and glycemia were the main parameters analyzed. Other factors affecting SUV were also reviewed to report potential confounding variables that could affect the relation of glycemia on 18F-FDG uptake.

### Synthesis of results and risk of bias

A meta-analysis was planned if the data from the included studies were considered relatively homogeneous. The effect of glycemia on SUV was evaluated by two statistics according to the data provided by each study: the coefficient of determination (R^2^) and the effect size (ES). Effect size was calculated as the difference between the mean SUV_max/mean_ of the control group (normoglycemic group) versus the mean SUV_max/mean_ of the hyperglycemic group divided by the pooled standard deviation. Herein, ES was defined as very small (<0.2), small (0.2–0.5), medium (0.5–0.8), large (0.8–1.2) and very large (1.2–2) [10]. If more than two hyperglycemic groups were reported in the study, we averaged the effect size of these groups. Risk of bias across studies would be only appraised if a meta-analysis were possible. There is no validated tool yet indicated to assess risk of bias among cross-sectional studies [9].

## Results

### Study characteristics

**Fig 1:**
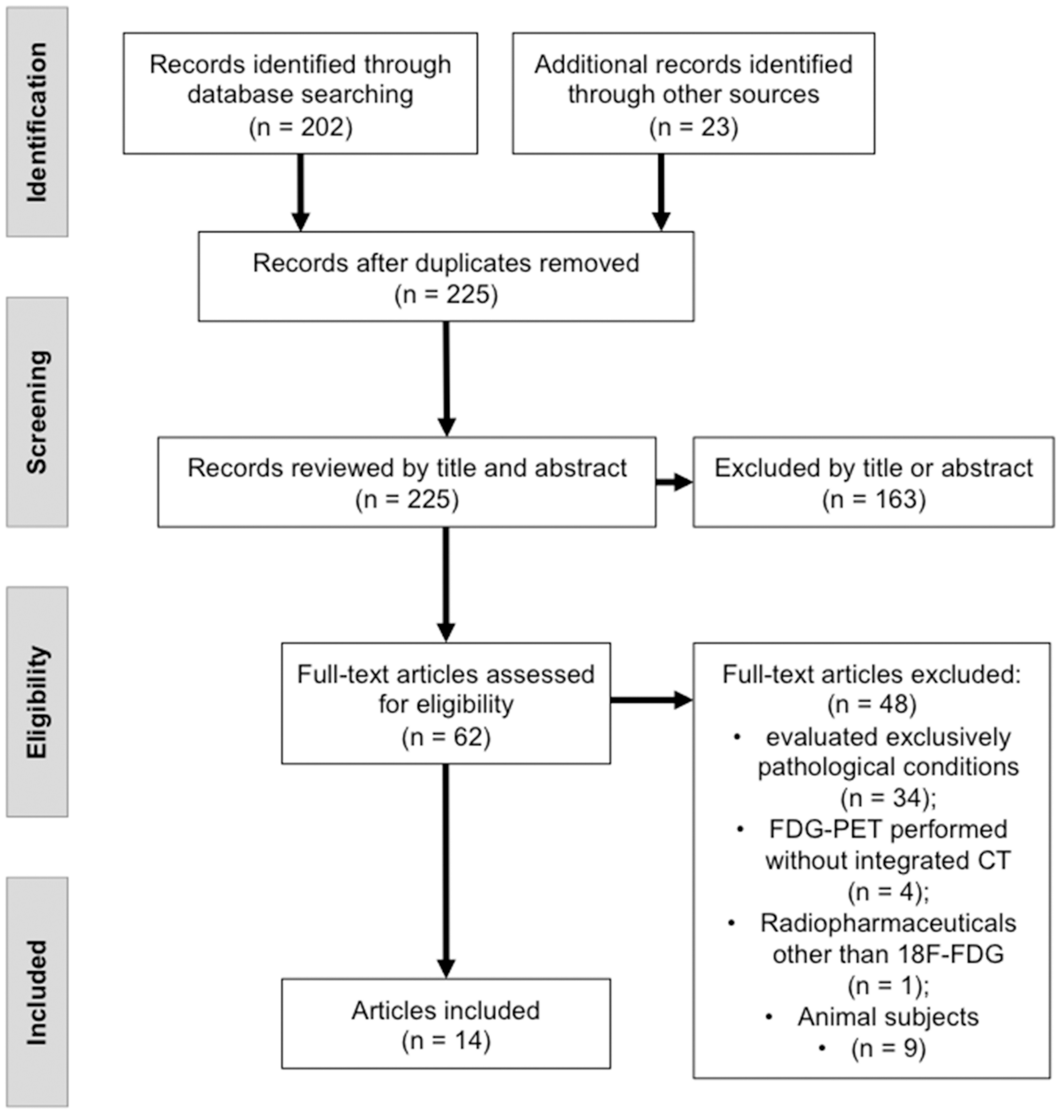
Preferred Reporting Items for Systematic Reviews and Meta-Analyses (PRISMA) flow diagram.

The literature search yielded 225 results, from which 62 full-text articles were evaluated and 14 met the inclusion criteria (Fig 1). Among the 225 results, 163 articles were excluded by title or abstract and 62 met the eligibility criteria for evaluation. The most common reason for exclusion among the analyzed papers was the evaluation of exclusively pathological conditions (n = 34 articles), animal studies (n = 9), FDG-PET performed without integrates CT scan (n = 4) and the use of radiopharmaceuticals other than FDG (n = 1). The literature search strategy is described in Fig 1.

The characteristics of the studies included are shown in Table 1. Nine evaluated the effect of different blood glucose levels on SUV measurements in multiple normal tissues [11–19], two exclusively in the liver [20,21], one exclusively in the brain[22] and two exclusively in the heart [23,24]. Nine studies were retrospective. Sample sizes were very heterogeneous, with a total of 2714 participants (mean, 193.8; range, 51–557). Most patients were referred for PET/CT due to various oncological indications.

**Table 1:** Study characteristics.

Study	Study Design	Sample (n)	Fasting duration	FDG dose (range or mean ± SD) (MBq)	Time of scanning (range)	Organs affected by glycemia (magnitude)	Not affected by glycemia	Factors affecting SUV (organ)^a^	Factors not affecting SUV
Büsing et al. 2013, Germany	R	DM (29) Insulin (22) Obese (28)	6h	248–393	77 min (45–124)	Brain (R^2^ = 0.19; ES = 1.26) Muscle (R^2^ = 0.05; ES = 0.83)	Liver, blood, tumor, spleen, lung, fat, heart	DM, Insulin (↑MSK, ↑fat, ↓brain), BMI (↑)	-
Lindholm et al. 2013, Sweden	R	High BG (62) vs. Normal BG (62)	N/A	4 MBq/kg	61 min (50–70)	Muscle (R^2^ = 0.05; ES = 0.51)	Liver, blood, BM, spleen, lung	DM, insulin (↑high BG group)	-
Webb et al. 2015, USA	R	BG<100 (53) BG 100–160 (149) BG 160–201 (27)	N/A	296–444	50 min	Liver (ES = 0.59)	Muscle, BM, tumor, heart	N/A	N/A
Malladi et al. 2012, USA	R	Oncological patients (557)	4-6h	462.5 ± 99.9	77.6 min (34–198)	Liver (R^2^ = 0.01) Blood (R^2^ = 0.02)	-	Age (↑), Male gender (↓), BMI (↑), glycemia (↑), incubation period (↓)	FDG dose, IV contrast, ethnicity
Kuruva et al. 2012, India	P	Oncological patients (88)	6h	N/A	76 min (±19.75)	-	Liver, blood	Incubation period (↓liver), weight (liver)	Age, gender, glycemia, DM
Groheux et al. 2013, France	P	Oncological patients (61)	N/A	5 MBq/kg	70 min (51–111)	Liver (R^2^ = 0.088)	Tumor	Age (↑), weight (↑)	Incubation period
Keramida et al. 2015, UK	R	BG <72 (35) BG 72–108 (156) BG >108 (35)	N/A	400 ±40	60 min	Liver (N/A) Brain (R^2^ = 0.58)	-	N/A	N/A
de Groot et al. 2004, Netherlands	R	Non-DM oncological patients (175)	13.0±4.2 h	200–220	60 min	-	Heart, bowel, stomach	-	Age, fasting period, glycemia
Viglianti et al. 2017, USA	R	Oncological patients (229)	N/A	466 ±12.6	N/A	Brain (N/A) Liver (N/A) Blood (N/A)	Spleen	DM (↑), BMI (↑)	Incubation period
Kubota et al. 2011, Japan	R	BG <125 (138)	6h	370	50–100 min	Liver (R^2^ = 0.062)	-	Incubation period (↑)	Age, gender
Mahmud et al. 2015, Malaysia	?	Oncological patients (51)	6h	327 ± 35.63	84 min (30–282)	Liver (R^2^ = 0.025)	-	BMI (↑), incubation period (↑), Age (↑)	FDG dose
Claeys et al. 2010, Belgium	R	Pediatrics (28) Adults non-DM (66)	6h	3.7 MBq/kg	60 min	Brain (N/A)	-	N/A	N/A
Kaneta et al. 2006, Japan	?	Oncological patients (159)	10.3 ±4.7 h	185	60 min	Heart (R^2^ = 0.03)	-	-	Age, fasting period
Israel et al. 2007, Israel	P	Oncological patients (504)	13 ±5 h	370–555	96 min (55–188)	Heart (ES 0.65)	-	↓: DM, bezafibrate, levothyroxine ↑: male gender, age (<30 y), fasting duration (<5h), heart failure, benzodiazepines	Insulin, BMI

BG = Blood glucose; BM = bone marrow; BMI = body mass index; DM = diabetes; MSK = Musculoskeletal; N/A = not available; P = prospective; R = retrospective; SD = standard deviation;? = unclear; R^2^ = coefficient of determination; ES = effect size; ↑ = positive correlation; ↓ = negative correlation

^a^ If not otherwise specified, all organs in the “positive correlation" are affected by the factor

Although the mean blood glucose level was reported in all studies (overall mean, 107.62 ± 16.29 mg/dl), stratification according to glycemic range for subgroup comparison was performed in only four studies [11–13,17]. Kubota el al. [20], Claeys et al. [22], Kaneta et al. [23], and de Groot et al.[18] did not include diabetic patients. In addition, 99% of patients included in the study conducted by Viglianti et al. were male [19].

### Study quality

The quality of the selected studies is shown in the table in S1 Table. We recorded a designation of “not applicable” (NA) when the response to the item was negative (e.g., no patient was excluded from the initial sample, no missing data were reported) or the item was not part of the study design (e.g., follow-up to gather further information). Meta-analysis was not performed, as the study samples were diverse (e.g., adult patients vs. pediatric patients), sampling stratification was different among studies (e.g., some used a glycemic range <100 mg/dl, whereas others used a cutoff of 150 mg/dl) and there was not a one single statistical summary measurement (e.g. effect size) to quantify the impact of hyperglycemia on SUVs.

### Imaging parameters

Imaging parameters reported varied considerably across articles. In six of them, subjects fasted for approximately 6 hours prior to 18F-FDG administration and PET/CT imaging. In three, fasting duration was >10 hours. However, for the remaining papers fasting duration could not be retrieved but can be presumed to follow existing guidelines.

Mean 18F-FDG dosages also ranged widely (185–466 MBq), and in some cases, unit conversion (milicurie to megabecquerel) had to be performed for comparison between studies. 18F-FDG dosage was reported as injected dose per body weight (MBq/kg) in three articles, and correlation could not be achieved in two of them, as mean body weight was not indicated [12,22]. Kuruva, et al. [15] did not specify mean 18F-FDG dose. Mean incubation time between 18F-FDG administration and PET/CT imaging was 69.34 min, but ranges were considerably wide in some studies (i.e., 30–282 min;[21] 34–198 min;[14] 55–188 min[24]).

The PET/CT scanners used were also different among studies. Seven used Siemens’s PET/CT scanners (Siemens Medical Solutions, Erlangen, Germany): Biograph 64 TruePoint TrueV [11,12,17,21], Biograph Sensation 16[20], Biograph T6[19], Biograph[23] and ECAT-ART[18]. Three reports used General Electric’s PET/CT scanners (GE Healthcare, Waukesha, WI USA): Discovery LS[24], Discovery ST[13] and Discovery STE [14]. Two papers adopted a Gemini XL PET/CT scanner (Philips Medical Systems, Netherlands) [16,20]. One of the studies did not specify which scanner was used [15].

### Synthesis of results

Most studies in this review evaluated the effect of blood glucose level on 18F-FDG uptake in one of two ways. Some researchers sought significant differences in mean SUV_max/mean_ between groups stratified by two or more glycemic ranges (eg, <150mg/dL vs. ≥150mg/dL). In this case, to objectively measure the magnitude of the association, we calculated the effect size (ES) of elevated blood glucose level in the hyperglycemic group compared with the lower glycemic range. Other researchers calculated Pearson’s coefficients (*r*) between glycemia and 18F-FDG uptake. In this case, we calculated the *R*^2^ values to measure the association of the variables. Results of organ-specific analysis of the association between glycemia and SUV_max/mean_ are shown in Table 2. The magnitude of this association and other factors affecting 18F-FDG uptake are shown in Table 1.

**Table 2:** Organ-specific analysis of the association between glycemia and SUVmax/mean.

	Büsing 2013	Lindholm 2013	Webb 2015	Malladi 2013	Kuruva 2012	Groheux 2013	Keramida 2015	Groot 2004	Viglianti 2017	Kubota 2011	Mahmud 2015	Claeys 2010	Kaneta 2006	Israel 2007
Brain	⊕						⊕		⊕			⊕		
Liver	-	-	⊕	⊕	-	⊕	⊕		⊕	⊕	⊕			
Blood pool	-	-		⊕	-				⊕					
Muscle	⊕	⊕	-											
Bone marrow		-	-											
Tumor	-		-			-								
Spleen	-	-							-					
Lung	-	-												
Fat	-													
Heart	-		-					-					⊕	⊕
Bowel								-						
Stomach								-						

⊕ = significant association, - = no association

### Effect of glycemia on the brain

Four authors reported a significant impact of glycemia on 18F-FDG uptake in the brains of patients with high blood glucose compared with those in lower glycemic ranges [11,17,19,22]. Subjects with higher glucose levels presented progressively lower SUV_max/mean_. The magnitude of the effect was very large and the variation in 18F-FDG uptake was well explained by the glycemic level (ES = 1.26; *R*^2^ = 0.16–0.58). Of note, the association between the blood glucose levels and SUV remained significant after controlling for potentially confounding factors, such as body mass index (BMI), injection to imaging time, and diabetes mellitus (DM) [19]. Therefore, the literature supports the dependence of brain SUVs on plasma blood glucose.

### Effect of glycemia on the liver

Most studies demonstrated a significant positive association between liver uptake and glycemia [13,14,16,17,19–21]. Few of these studies, however, reported specific measurements representing the impact of hyperglycemia on hepatic 18F-FDG uptake (ES = 0.59; *R*^2^ = 0.01–0.08). Busing[11] and Lindholm[12] showed non-significant trends (ES = 0.42 and ES = 0.24, respectively) toward a weak positive association between glycemia and the liver SUV. Viglianti et al [19] performed a multivariate analysis to explore this relation. The authors graphically reported a significant association between the plasma blood glucose level and liver SUV after adjustment for other variables; however, a quantitative measurement was not reported, only the graphical representation. In the graph, the impact of this relation appears to be very small. Malladi et al [14] reported similar findings based on multivariate analysis. In summary, 18F-FDG uptake in the liver is affected by the glycemic levels, but the magnitude of this effect is small.

### Effect of glycemia on the mediastinal blood pool

Only two of five studies showed a significant influence of glycemia on the blood-pool SUV, as determined by multivariate analyses [11,12,14,15,19]. However, the impact of glycemia was demonstrated to be very small in both studies (*R*^2^ = 0.02) [14,19]. Kuruva et al. [15] found no significant association between the variables also in a multivariate analysis. Thus, the effect of glycemia on the mediastinum appears to be negligible.

### Effect of glycemia on other tissues

Two authors reported a small to moderate positive effects of hyperglycemia on the muscles (ES = 0.51–0.83; *R*^2^ = 0.05) [10,11]. Webb et al. [13] found a small non-significant trend (ES = 0.23, p = 0.055) toward association between these variables. Hyperglycemia does not appear to directly affect myocardial 18F-FDG uptake. [11,13,24] Israel et al. [24] found a moderate effect of glycemia ≥150 mg/dl on the myocardial SUV (ES = 0.65); however, this effect disappeared when other variables (e.g., DM, age, sex) were considered in the multivariate analysis. Lung [11,12], bone marrow [12,13], tumor [11,13,16], spleen [11,12,19], fat [11], bowel [18], and stomach [18], 18F-FDG uptake were not influenced by blood glucose levels.

### Influence of other factors on 18F-FDG uptake

Many factors other than blood glucose were shown to influence 18F-FDG uptake. However, different studies have yielded conflicting results regarding the impacts of these variables (Table 1). Notably, that the effect of each factor appears to be organ specific. For instance, diabetic patients were shown to have significantly higher muscle and fat SUVs, but lower brain SUVs when compared to non-diabetics, while the liver and mediastinum were minimally affected [11,19].

Most studies involving multivariate analyses with adjustment showed that BMI, age, and diabetes status appeared to affect 18F-FDG uptake [14–16,19,21]. Four of five studies that investigated the relation of BMI to SUV showed that BMI has an important impact on 18F-FDG uptake [11,14,19,21]. The heart was the only organ in which the BMI was not associated with the SUV in a multivariate analysis [24]. The impact of the incubation period remains unclear, as some studies have reported a positive significant relationship, whereas others pointed in the opposite direction (Table 1). In contrast, the 18F-FDG dose did not appear to influence uptake [14,21].

## Discussion

This literature review indicated that the brain is the only organ in which hyperglycemia has a large effect on the SUV. Although the liver and mediastinal blood pool are significantly impacted by glycemia, these effects appear to be too small to be of clinical relevance. The lung, bone marrow, tumor, spleen, fat, bowel, and stomach were not found to be influenced by the plasma glucose levels. Other factors, such as BMI, age and diabetes status, were also shown to affect 18F-FDG uptake; thus, they should be taken into account in future studies of the effects of blood glucose levels on the SUVs of different organs.

Whereas many variables other than glycemia, such as sex, BMI, and age, play important roles in the 18F-FDG uptake, they are not likely to vary between PET/CT studies in individual subjects. On the other hand, the fasting plasma glucose levels may vary significantly between examinations, especially in diabetic patients [16,25]. In light of this variation, many authors have reported alternatives to reduce weight and glycemia dependence in SUV analysis, such as corrections for lean body mass (SUL) and body surface area (SUVbsa) [1,26–28]. Moreover, the PET Response Criteria in Solid Tumors (PERCIST) recommend the use of liver SUL over SUV_max_ and glucose correction as a reference for the definition of disease and assessment of therapy response, as it has less test-retest variance [5]. Wahl et al.[5] emphasized that the metabolic response should be evaluated only when the liver is free of disease and the absolute difference in SUVs obtained initial and subsequent studies is <0.3. Otherwise, the mediastinal blood pool should be used as the reference tissue.

Contrary to the PERCIST guidelines, our review suggests that the blood pool is a more optimal reference tissue than the liver, as it is much less dependent than the liver on glycemia and other variables [19]. However, other tissues, such as the lung, must be considered as background organs in future guidelines, as such tissues have been shown to be unaffected by glycemia and their measurement is less difficult than that for the mediastinal blood pool, which involves the drawing of ROIs at multiple levels of the aorta. Additionally, despite current recommendations to reschedule imaging studies for patients with glycemia >200 mg/dl [2,3], our review suggests that such rescheduling is not necessary. Although an association between 18F-FDG uptake and the liver SUV exists, its impact is too small to be clinically relevant for tumor diagnosis or treatment follow-up.

The current recommendation for brain PET/CT is to limit 18F-FDG administration in patients with plasma blood glucose levels <160 mg/dl [4]. However, our review revealed greater variation in brain SUV in the glycemic range of <130 mg/dl. For instance, doubling of glycemia from 60 mg/dl to 120 mg/dl results in a reduction in 18F-FDG uptake of almost 50% [19]. Taking this finding into consideration, recent studies demonstrated that brain imaging in healthy volunteers with hyperglycemia could reveal patterns that are similar to the findings for neurodegenerative diseases [6]. Moreover, the brain SUV appears to be correlated with glycemia even after normalization to blood glucose [17], although one author found that glucose corrections improved the accuracy of high-grade gliomas diagnosis [29]. Thus, future studies should focus on better normalization options, such as the use of values from other internal organs, to improve the value of brain PET/CT for the early diagnosis of neurodegenerative diseases in populations with elevated glycemic levels.

The effect of unlabeled serum glucose on 18F-FDG uptake is more pronounced in tissues with higher glucose metabolism, such as the brain, but the underlying mechanism of this relationship is not completely understood [11]. In the brain, as 18F-FDG enters the cells using the same saturable glucose transporter as unlabeled glucose, glucose elevation should result in decreased 18F-FDG uptake [11,22]. However, this mechanism is more complex than just competitive inhibition alone, as demonstrated by the nonlinear, but rather exponential, relationship between glycemia and the SUV [19]. Observations in rats demonstrated that the brain behaves in a transporter-limited fashion in the hypoglycemic to euglycemic range, but then switches to an intracellular phosphorylation-limited process in the hyperglycemic state [19,30].

Technological factors, such as inter-scanner variability, image acquisition, reconstruction parameters, and variability between readers, might also have substantial impacts on SUV measurement [31], and these variables were considerably heterogeneous across the studies analyzed in this review. Although authors reported using devices made by only three manufacturers (Siemens, General Electric, and Philips), eight different PET/CT scanner models were used in the included studies. Due to differences in physical properties, such as heterogeneous detector crystal dimensions, and acquisition and reconstruction options, such as matrix size, field of view, and time-of-flight, SUV may vary by up to 22.6% among scanners [32].

The limitations of our review included those attributable to the study design; the small number of papers evaluating organs such as the heart, muscle and blood pool; and the differences of the statistical reporting on the effects of glycemia on FDG uptake among studies. In addition, the studies were very diverse in terms of scanner type, FDG dose injected, timing to scan, and inter-reader variability, which may have contributed to variations in the results. Despite these limitations, the findings of this review are likely applicable to a more diverse patient population.

## Conclusions

This review showed that the impact of glycemia on the 18-F-FDG uptake in most tissues, except the brain, is negligible or too small to be clinically significant. Future studies should explore the use of other background tissues that are less affected by other factors, such as BMI, and seek better normalization methods for the brain.

## Supporting information

S1 File Prisma checklist. (PDF)

S2 File Search strategy in MEDLINE database. (PDF)

S1 Table Agency for Healthcare Research and Quality (AHRQ) checklist to assess quality of the included studies. (PDF)
