# Supplementary material for: Effects of blood glucose level on 18F-FDG uptake for PET/CT in normal organs: A systematic review
Source: PLoS One. 2018 Feb 27;13(2):e0193140. doi: 10.1371/journal.pone.0193140 (PMC5828444; doi:10.1371/journal.pone.0193140)
Supplement: S2 File — (PDF) [file pone.0193140.s002.pdf]

S2 File. Search strategy in MEDLINE database

(SUV[All Fields] AND (“blood glucose”[MeSH Terms] OR (“blood”[All Fields] AND “glucose”[All Fields]) OR “blood glucose”[All Fields] OR “glycemia”[All Fields])) AND (normal[All Fields] OR healthy[All Fields]).
