# Supplementary material for: Effects of blood glucose level on 18F-FDG uptake for PET/CT in normal organs: A systematic review
Source: PLoS One. 2018 Feb 27;13(2):e0193140. doi: 10.1371/journal.pone.0193140 (PMC5828444; doi:10.1371/journal.pone.0193140)
Supplement: S1 Table — (PDF) [file pone.0193140.s003.pdf]

S2 Table. Agency for Healthcare Research and Quality (AHRQ) checklist to assess quality of the included studies

ARHQ Methodology Checklist for Cross-sectional study (http://www.ncbi.nlm.nih.gov/books/NBK35156/)	Büsing KA, et al. 2013	Lindholm, et al. 2013	Webb, et al. 2015	Malladi, et al. 2013	Kuruva, et al. 2012	Groheux, et al. 2013	Keramida, et al. 2015	Groot, et al. 2004	Viglianti, et al. 2017	Kubota, et al. 2011	Mahmud, et al. 2015	Claeys, et al. 2010	Kaneta, et al. 2006	Israel et al, 2007
1. Define source of information (survey, record review)	⊕	⊕	⊕	⊕	⊕	⊕	⊕	⊕	⊕	⊕	⊕	⊕	⊕	⊕
2. List inclusion and exclusion criteria for exposed and unexposed subjects (cases and controls) or refer to previous publications	⊕	⊕	⊕	⊕	⊕	⊕	⊕	⊕	⊕	⊕	⊕	⊕	⊕	⊕
3. Indicate time period used for identifying patients	⊕	⊕	-	⊕	-	-	⊕	-	⊕	⊕	⊕	-	-	-
4. Indicate whether or not subjects were consecutive if not population-based	U	⊕	⊕	U	U	U	⊕	U	⊕	⊕	U	U	U	⊕
5. Indicate if evaluators of subjective components of study were masked to other aspects of the status of the participants	-	-	-	-	-	-	-	-	-	-	-	-	-	-

6. Describe any assessments undertaken for quality assurance purposes (e.g., test/retest of primary outcome measurements)	⊕	⊕	⊕	⊕	⊕	-	⊕	-	-	⊕	⊕	⊕	-	⊕
7. Explain any patient exclusions from analysis	⊕	⊕	NA	NA	⊕	NA	NA	NA	⊕	⊕	⊕	NA	NA	⊕
8. Describe how confounding was assessed and/or controlled	U	-	-	⊕	-	-	-	U	⊕	⊕	⊕	-	-	⊕
9. If applicable, explain how missing data were handled in the analysis	NA	⊕	NA	NA	NA	NA	-	NA	NA	NA	NA	NA	NA	⊕
10. Summarize patient response rates and completeness of data collection	⊕	⊕	⊕	⊕	⊕	⊕	-	U	⊕	⊕	⊕	U	-	⊕
11. Clarify what follow-up, if any, was expected and the percentage of patients for which incomplete data or follow-up was obtained	NA	NA	NA	NA	NA	NA	NA	NA	NA	NA	NA	NA	NA	NA

Yes = ⊕; No = -; Unclear = U; Not applicable = NA
